# Written Recommendation to Repeat Colonoscopy within 1 Year after Inadequate Bowel Preparation: A Simple and Highly Effective Intervention

**DOI:** 10.1055/a-2973-7245

**Published:** 2026-10-16

**Authors:** Marta Antón Arnal, Nadine Grima Sopesens, Agustín Seoane Urgorri, Xavier Duran Jorda, Marc Batlle Garcia, Miriam Parrilla Carrasco, Eva Pont Feijoo, Anna Mañé Bosch, Jordi Muñoz Galito, Faust Riu Pons, Josep Maria Dedeu Cuscó, Xavier Bessa Caserras, Luis Barranco Priego, Miguel Ángel Pantaleón Sánchez

**Affiliations:** 1 Department of Gastroenterology 16548 Hospital del MarBarcelonaSpain; 2 16551 Hospital del Mar Medical Research InstituteBarcelonaSpain; 3 117357 Universitat Pompeu Fabra Departament de Medicina i Ciencies de la VidaBarcelonaSpain; 4 Medical Statistics Unit, MARData, Scientific & Technical Services 16551 Hospital del Mar Medical Research InstituteBarcelonaSpain

**Keywords:** inadequate bowel preparation, colonoscopy, written recommendation

## Abstract

**Background and Study Aim** When bowel preparation is inadequate, international guidelines recommend repeating the colonoscopy within 1 year. We aimed to evaluate the effectiveness of providing a written recommendation for improving completion of repeat colonoscopy within 1 year after inadequate bowel preparation.

**Materials and Methods** We conducted a single-center, nonrandomized clinical trial. Consecutive outpatients’ colonoscopies with inadequate bowel preparation were included. The intervention group received written recommendations concerning the necessity of repeating the colonoscopy within 1 year. Both groups were provided with verbal instructions conveying the same information. Inadequate bowel preparation was defined as a Boston Bowel Preparation Scale score of <2 points in any segment.

**Results** In total, 464 patients with inadequate bowel preparation were included. Adherence to repeat colonoscopy within 1 year was significantly higher in the intervention group compared with the control group (54.4% vs. 29.4%; *p* < 0.001). On repeat colonoscopy, the adenoma detection rate was 31.5% and 1.5% of patients who were found to have colorectal cancer during this procedure. In the multivariate analysis, provision of a written recommendation was the most strongly associated factor with completion of repeat colonoscopy within 1 year (OR 3.40; 95% CI 2.22–5.26; *p* < 0.001).

**Conclusion** Providing a written recommendation following inadequate bowel preparation improves completion of repeat colonoscopy within 1 year. Implementing a mandatory field in the colonoscopy report is an effective strategy to ensure patients undergo repeat colonoscopy in accordance with clinical guidelines (NCT05709145).

## Introduction

Bowel preparation quality is critical for the efficacy of colonoscopy. 1 However, up to one-quarter of colonoscopies fail bowel cleansing. 2 Suboptimal index exam independently increases colorectal cancer (CRC) incidence and mortality. 3 That is why international guidelines issue strong recommendations for a 1-year repeat colonoscopy in case of inadequate bowel preparation. 4 5

Completion of recommended follow-up colonoscopy is crucial to ensure the efficiency of colonoscopy and depends on both clinician-related processes and patient attendance. A key reason for inadequate surveillance is the inappropriate recommendations given by health care providers. 6 Limited evidence indicates highly variable follow-up recommendations in cases of inadequate bowel preparation, resulting in a low compliance for a 1-year repeat colonoscopy. 7 A multicenter study involving over 250,000 colonoscopies revealed that only one-third of the colonoscopies included a written recommendation for the need for repeat colonoscopy within 1 year. 8 Another study with 9170 patients showed that this 1-year-recommendation was included in only 10% of colonoscopy reports, with 65% of patients being advised to repeat the procedure in 4 or more years. In contrast, for colonoscopies with optimal preparation, most recommendations (95%) were concordant with the guidelines. 9 There is a clear need to expand written recommendations in case of inadequate bowel preparation to avoid missing clinically relevant lesions. These studies highlight that follow-up recommendations provided by physicians are, at best, inappropriate, and, at worst, absent. Of note, no studies to date have specifically addressed the usefulness of written recommendations in improving adherence to guideline-concordant follow-up intervals in real-world clinical practice.

The aim of this study was to evaluate the effectiveness of written recommendations following inadequate bowel preparation in improving completion of repeat colonoscopy within 1 year.

## Materials and Methods

### Study Design

A single-center, nonrandomized clinical trial was conducted to determine whether a written recommendation could increase completion of repeat colonoscopy within 1 year following inadequate bowel preparation. The study included a retrospective cohort without written recommendations, which was the control group. A prospective cohort received written recommendations to repeat the colonoscopy within 1 year, thereby constituting the intervention group. Both groups received oral instructions regarding the appropriate surveillance interval after the index colonoscopy.

The study was conducted between January 2021 and December 2023. The study protocol was approved by the Ethics Committee for Research Involving Medicinal Products of Parc de Salut MAR (Hospital del Mar, Approval Reference No. 2023/10823/I). The investigation adhered to the ethical principles outlined in the Declaration of Helsinki and was registered at ClinicalTrials.gov (NCT05709145).

### Study Population

The study population comprised consecutive outpatients who underwent colonoscopy at our center with inadequate bowel preparation. Patients undergoing colonoscopy for either symptomatic evaluation or surveillance were included. Screening colonoscopies, which follow special circuits involving in-person visits to our hospital, were excluded from the study. Additionally, colonoscopies scheduled through special reprogramming circuits, such as for complex polyps or piecemeal endoscopic resection of polyps ≥20 mm, as well as those involving hospitalized patients, were also excluded.

### Control Group

Consecutive patients with inadequate bowel preparation colonoscopy were included retrospectively from 1 January to 31 December 2021. All patients were instructed orally to repeat the colonoscopy after the procedure. Although the colonoscopy reports documented inadequate bowel cleansing, no written recommendation was provided regarding the necessity and timing of the repeat colonoscopy.

### Intervention Group

Consecutive patients with inadequate bowel preparation colonoscopy were included prospectively from 1 January to 31 December 2023. All patients received written recommendations in the colonoscopy report on the need for repeat colonoscopy within 1 year due to inadequate bowel cleansing. They also received the same information orally after procedure.

### Written Recommendation Intervention

To ensure that all patients in the intervention group received the written recommendation, a mandatory bowel preparation tab was created in the electronic colonoscopy report (Endobase, Olympus). When inadequate bowel preparation was clicked, the following text was automatically included in the colonoscopy report: “The colonoscopy is invalid due to inadequate bowel preparation. It is recommended to repeat the colonoscopy in less than 1 year” ( **Appendix,** **Supplemental Figs. 1 and 2, available in the online version** ).

### Outcome Measures and Definitions

The primary outcome was completion of repeat colonoscopy within 1 year as established by international guidelines in case of inadequate bowel preparation.

Inadequate bowel preparation was defined as a Boston Bowel Preparation Scale (BBPS) score of less than 2 points in any colonic segment. Endoscopists assessed the BBPS after completion of all cleansing maneuvers.

The index colonoscopy was defined as the initial colonoscopy with inadequate bowel preparation, and the repeat colonoscopy as a subsequent examination performed within 1 year.

Physicians who requested the index colonoscopy were also responsible for requesting the repeat colonoscopy, either following the written recommendations or, if no recommendation was provided, based on their clinical judgment. No repeat colonoscopy was scheduled directly by the endoscopy unit.

We assessed the diagnostic yield of repeat colonoscopy for the detection of new lesions, considering only valid procedures (with both adequate bowel preparation and successful cecal intubation). For the repeat colonoscopies, we considered only newly detected lesions; previously detected lesions or recurrence after polypectomy were excluded.

### Statistics

The sample size calculation was based on institutional data, given the limited published evidence on completion of repeat colonoscopy after inadequate bowel preparation and the lack of studies specifically assessing the effect of a written recommendation. In 2021, completion of repeat colonoscopy within 1 year was 28% (87/309) among patients who did not receive a systematic written recommendation and 57.9% (22/38) among those who did. To avoid overestimating the expected effect of the intervention, we conservatively assumed completion rates of 30% in the control group and 45% in the intervention group. The sample size calculation was performed using the GRANMO sample size calculator (version 8.0) and was based on a two-sided Z-test comparing two independent proportions. Assuming a two-sided significance level of 0.05, 80% power, and an anticipated loss to follow-up of 5%, a minimum of 171 patients was required in the intervention cohort. For the historical control cohort, all consecutive eligible patients from the entire 2021 calendar year were included, resulting in 293 controls. Retaining all eligible controls increased statistical precision while making use of all available data. The intervention cohort was prospectively closed after reaching the planned sample size.

Categorical variables were compared using Pearson’s chi-squared or Fisher’s exact test, and continuous variables using Student’s *t* test. Two-tailed *P* values < 0.05 were considered statistically significant. Analyses were performed using Stata 15.1 (StataCorp LLC, College Station, TX, USA).

Univariate and multivariable logistic regression analyses were performed to assess factors associated with completion of repeat colonoscopy within 1 year. Candidate variables were selected a priori based on previous literature and on clinically plausible patient-, procedure-, and endoscopist-related factors. These included intervention group, polyp detection at index colonoscopy, incomplete index colonoscopy, indication for colonoscopy, index colonoscopy requested by a gastroenterologist, previous colonoscopy, and sex. Variables associated with the outcome in the univariate analysis were subsequently entered into the multivariable model. Results from univariate analyses are presented as crude odds ratios (cORs), whereas results from the multivariable model are presented as adjusted odds ratios (aORs), both with 95% confidence intervals (CIs).

## Results

### Patient Characteristics

The study included a total of 464 patients: 293 in the control group, without written recommendation, and 171 in the intervention group, adding a written recommendation in the colonoscopy report. In the control group, the median age was 62.3 years, 51.2% were men, and 66.9% of patients were undergoing colonoscopy because of symptoms. In the intervention group, the median age was 62.4 years, 55% were men, and 75.4% of patients were undergoing colonoscopy because of symptoms. There were no significant differences in any of the baseline characteristics of the patients between the two groups ( Table 1 ).

**Table 1:** Baseline characteristics of the patients regarding index colonoscopy.

Variable	Control ( *n* = 293)	Intervention ( *n* = 171)	*p* -Value
Male sex, *n* (%)	150 (51.2)	94 (55)	0.43
Age, mean (SD), years	62.3 (15.6)	62.4 (15.3)	0.96
BMI, mean (SD)	27.3 (5.8)	29.7 (28.8)	0.16
Language barrier, *n* (%)	30 (10.2)	15 (8.8)	0.60
Prior endoscopy, *n* (%)	158 (53.9)	89 (52.0)	0.69
Prior unattendance, *n* (%)	57 (19.5)	41 (24.0)	0.25
Personal history of colorectal cancer, *n* (%)	13 (4.4)	14 (8.2)	0.09
ASA classification, *n* (%)			0.34
I	54 (18.4)	16 (9.4)	
II	151(51.5)	109 (63.7)	
III	85 (29.0)	44 (25.7)	
IV	3 (1.0)	2 (1.2)	
Abdominal/pelvic surgery, *n* (%)	98 (33.4)	58 (33.9)	0.91
Constipation, *n* (%)	62 (21.2)	43 (25.1)	0.32
Opiates, *n* (%)	19 (6.5)	17 (9.9)	0.17
Tricyclic antidepressants, *n* (%)	5 (1.7)	5 (2.9)	0.38
Calcium blockers, *n* (%)	26 (8.9)	25 (14.6)	0.56
Diabetes mellitus, *n* (%)	72 (24.6)	48 (28.1)	0.40
Outpatient colonoscopy, *n* (%)	228 (77.8)	118 (69.0)	0.36
Request by gastroenterologist, *n* (%)	57 (19.5)	27 (15.8)	0.31
Indication, *n* (%)			0.51
Symptoms	196 (66.9)	129 (75.4)	
Surveillance	87 (29.7)	38 (22.2)	
Family screening	10 (3.4)	4 (2.3)	
Staff endoscopist, *n* (%)	156 (53.1)	91 (53.2)	0.97
AM endoscopy schedule, *n* (%)	139 (47.6)	66 (38.6)	0.06

Abbreviations: SD, standard deviation; BMI, body mass index; ASA, American Society of Anesthesiologists.

### Primary Outcome: Completion of Repeat Colonoscopy

A significantly higher proportion of patients in the intervention group underwent a repeat colonoscopy within 1 year after an inadequately prepared index procedure, compared with the control group (54.4% vs 29.4%, *p* < 0.001) ( Table 2 ).

**Table 2:** Performance of repeat colonoscopy within 1 year after a colonoscopy with inadequate bowel preparation.

Group	Colonoscopy repeated within 1 year, *n* (%)	95% CI
Control ( *n* = 293)	86 (29.4)	24.2–35.0
Intervention ( *n* = 171)	93 (54.4)	46.6–62.0
*p* -Value	–	<0.001

Abbreviation: CI, confidence interval.

### Secondary Outcomes

#### Repeat Colonoscopy Outcomes

All patients (100%) in the intervention group received written recommendations in their colonoscopy report regarding the need to undergo a repeat colonoscopy within 1 year. Patients in the intervention group were significantly more likely to have a repeat colonoscopy requested by the referring physician compared with the control group (61.4% vs 35.5%; *p* < 0.001). No-show was defined as failure to attend a scheduled repeat colonoscopy after referral. The no-show rate for repeat colonoscopy was 16.3% in the control group and 11.4% in the intervention group ( *p* = 0.3). A valid colonoscopy was achieved in 130 repeat procedures including both groups, 64.0% of the control group and in 80.6% of the intervention group ( *p* = 0.012) ( Table 3 ).

**Table 3:** Outcomes related to repeat colonoscopy.

	Control ( *n* = 293)	Intervention ( *n* = 171)	*p* -Value
Written recommendation, *n* (%)	0 (0)	171 (100)	<0.001
Repeat colonoscopy requested by referring physician, *n* (%)	104 (35.5)	105 (61.4)	<0.001
No-show to repeat colonoscopy < 1 year, *n* (% of those requested)	18/104 (17.3%)	12/105 (11.4%)	0.226
Valid repeat colonoscopy, *n* (% of those attending)	55/86 (64.0%)	85/93 (80.6%)	0.012

#### Lesion Detection in Repeat Colonoscopy

At the valid repeat colonoscopies, including patients from both the control and intervention groups, the polyp detection rate was 43.1% (95% CI 34.5–52.1) and the adenoma detection rate (ADR) was 31.5% (95% CI 23.6–40.2). CRC was discovered in 2 patients (1.5%; 95% CI 0.2–5.4%). There were no differences in the adenoma, polyp or CRC detection rate between groups ( Table 4 ).

**Table 4:** Lesion detection rate in valid repeat colonoscopies.

Lesion	Global ( *n* = 130)	95% CI
Polyp, *n* (%)	56 (43.1)	34.5–52.1
Adenoma, *n* (%)	41 (31.5)	23.6–40.2
CRC, *n* (%)	2 (1.5)	0.2–5.4

Abbreviations: CRC, colorectal cancer; CI, confidence interval.

#### Factors Associated with Completion of Repeat Colonoscopy within 1 Year

Univariate analysis identified several factors associated with completion of repeat colonoscopy within 1 year: inclusion of a written recommendation (OR 2.87; 95% 1.94–4.26, *p* < 0.001), incomplete index colonoscopy (OR 2.62; 95% 1.78–3.88, *p* < 0.001), surveillance colonoscopy (OR 2.18, 95% 1.43–3.32, *p* < 0.001) and having a previous colonoscopy (OR 1.79; 1.22–2.63, *p* = 0.003).

Multivariable analysis showed that the provision of a written recommendation for follow-up was the most strongly associated factor with completion of repeat colonoscopy within 1 year (adjusted OR 3.40; 95% CI 2.22–5.26; *p* < 0.001). Additionally, having an incomplete index colonoscopy (adjusted OR 2.56; 95% CI 1.66–4.00; *p* < 0.001) and an indication for surveillance colonoscopy (adjusted OR 2.77; 95% CI 1.66–4.68; *p* < 0.001) were associated with a significantly increased compliance to repeat colonoscopy within 1 year ( Table 5 ). No other patient, endoscopist, or procedure-related characteristics were associated with completion of repeat colonoscopy within 1 year.

**Table 5:** Univariate and multivariate analyses.

Variable	Univariate		Multivariate	
	OR (95% CI)	*p* -Value	OR (95% CI)	*p* -Value
Intervention group	2.87 (1.94–4.26)	<0.001	3.40 (2.22–5.26)	<0.001
Polyp detection in index colonoscopy	0.55 (0.34–0.87)	0.013	0.59 (0.33–1.03)	0.066
Incomplete index colonoscopy	2.62 (1.78–3.88)	<0.001	2.56 (1.66–4.00)	<0.001
Indication				
Symptoms	–	–	–	–
Surveillance	2.18 (1.43–3.32)	<0.001	2.77 (1.66–4.68)	<0.001
Family screening	1.51 (0.49–4.44)	0.458	2.30 (0.61–8.30)	0.204
Request by gastroenterologist	1.77 (1.10–2.85)	0.019	1.39 (0.81–2.39)	0.234
Previous colonoscopy	1.79 (1.22–2.63)	0.003	1.38 (0.87–2.18)	0.167
Sex (female)	0.72 (0.49–1.05)	0.091	0.81 (0.53–1.23)	0.329

Abbreviations: OR, odds ratio; CI, confidence interval.

## Discussion

We found that providing a written recommendation for follow-up after inadequate bowel preparation significantly increases completion of repeat colonoscopy within 1 year. Our findings underscore the pivotal role of the endoscopist in offering written follow-up guidance, as recommended by international guidelines. 4 10 These results are novel, as no previous study has evaluated the impact of a written recommendation on adherence to follow-up in clinical practice outside of a screening setting.

Completion of repeat colonoscopy in the intervention group was markedly higher than in the control group (54.4% vs 29.4%, *p* < 0.001). There is little evidence evaluating the impact of written recommendations on completion of repeat colonoscopy. In a recent study, 59.2% who were recommended for follow-up colonoscopy underwent the procedure within 1 year. These results are consistent with ours; however, their study had some limitations: it was a retrospective single-center study conducted in a predominantly white male veteran population, which limits external validity. Additionally, it lacked a control group, used a non-validated bowel preparation scale, included only screening colonoscopies, and excluded symptomatic patients—who may be less likely to return if symptoms have resolved. Another retrospective study showed that only up to 15–19% repeat the procedure within 1 year, which is substantially lower than the completion rate observed in our study. In this study, 17 patients were instructed to have next-day repeat colonoscopy and were significantly more likely to adhere to the recommendation (47%) compared to longer intervals. 7 However, the small sample size and the operational demands of scheduling next-day procedures—requiring flexibility from the endoscopy unit and the patient—limit the feasibility and generalizability of this approach. 11 Overall, the evidence on the efficacy of a next-day colonoscopy compared with a later colonoscopy is limited and conflicting. 4 Our findings demonstrate that a simple, structured intervention can substantially improve completion of repeat colonoscopy, highlighting its potential value in routine clinical practice.

We achieved a 100% written recommendation guideline-concordant in the intervention group thanks to the inclusion of a mandatory automatic tab in case of inadequate bowel preparation ( **Appendix** , **Supplemental Figs. 1 and 2, available in the online version** ). Strong recommendations for a 1-year repeat colonoscopy when bowel preparation fails were issued by international guidelines. 4 5 Despite strong recommendations, limited evidence suggests that guideline-concordant recommendation is surprisingly inconsistent, with highly variable follow-up interval recommendations after inadequate bowel preparation. In a colonoscopy registry of 9170 average risk patients, only 10% of screening colonoscopies with poor preparation were advised to repeat within 1 year, and 71.5% were given a follow-up of 4 or more years. 9 In a most recent study among 260,314 colonoscopies with inadequate bowel preparation, the recommendation for follow-up interval within 1 year was given in 31.9% – rising from 21.0% before to 34.4% after the guideline’s publication. 8 Another study showed 48.5% of colonoscopies received a recommendation for repetition within 1 year. 7 However, the aforementioned have several flaws: (1) colonoscopies with inadequate bowel preparation represented a small subset of the study populations, (2) most were retrospective, (3) relied on subjective bowel preparation scales, (4) focused primarily on screening populations, and (5) predated guideline publication. In contrast, our study prospectively targeted patients with inadequate bowel preparation and implemented a structured intervention after guideline publication. Our findings indicate that automating the insertion of a written recommendation for follow-up eliminates the first barrier to completion: the variability and subjectivity in the endoscopist’s recommendation. Whether due to ignorance or forgetfulness, the human factor can generate inaccurate recommendations, preventing the patient from attending the procedure within the interval stipulated by the guidelines. Our intervention ensured that all endoscopists—regardless of experience, or findings in index colonoscopy—consistently issued guideline-concordant instructions. Guidelines recommend that the endoscopy unit should advise the patient on the appropriate surveillance interval with both written and oral instructions. 10 Although this is also a strong recommendation, it is based on low-quality evidence. A barrier for the implementation of this recommendation is the unavailability of histologic results at the time of the report. However, in cases of inadequate preparation, as it is an invalid procedure per-se, this barrier is absent allowing the follow-up interval to be communicated immediately. Our intervention demonstrated that systematic integration of decision-support tools can fully align clinical practice with evidence-based recommendations.

An important consideration is that our primary outcome should not be interpreted as a pure measure of patient adherence. In our setting, repeat colonoscopy could not be directly scheduled by the endoscopy unit because local administrative and reimbursement procedures require a new request from the referring physician after review of the colonoscopy report. Therefore, completion of repeat colonoscopy within 1 year reflects a multistep process involving both physician behavior and patient follow-through. In this context, the bottleneck in scheduling a repeat colonoscopy lies in the physician’s request during the post-colonoscopy consultation. In our study, written recommendations significantly increase the likelihood that the referring physician will request a repeat colonoscopy (61.4% vs 35.5%; *p* < 0.001). It is essential that the colonoscopy reports include the elements necessary for a high-quality report 12 13 ; however, there is variation in the clarity and content of the endoscopist’s post-colonoscopy communication. 14 15 The proportion of patients scheduled for a repeat colonoscopy beyond 1 year was higher in the control group (22.4%) compared with the intervention group (12.0%), with the difference lying at the threshold of statistical significance ( *p* = 0.05). This finding may reflect that primary care physicians are often unfamiliar with the exact surveillance intervals recommended in current guidelines. In such cases, the decision to schedule a repeat examination may be made somewhat arbitrarily or based on general clinical judgment rather than the guideline-specific timeframe. A recent study highlighted that clear and precise guidance on the indication and timing of repeat colonoscopy is considered a key priority among general practitioners. 16 Providing this written endoscopist recommendation indicating the steps to be taken could reduce the risk of missed or delayed care. In other healthcare systems, direct ordering or early rescheduling by the endoscopy unit could represent an additional strategy to improve completion of guideline-recommended repeat colonoscopy.

No-shows to scheduled repeat colonoscopy appointments negatively affect completion of recommended follow-up procedures. In our study, the no-show rate for repeat colonoscopy was numerically higher in the control group than in the intervention group (16.3% vs 11.4%, *p* = 0.3). Providing written recommendations may reinforce the physician’s verbal advice and emphasize the importance of undergoing the repeat examination. However, the small sample size (17 and 12 patients in each group, respectively) substantially limits the statistical power to detect meaningful differences. Data on no-shows among patients scheduled for a repeat colonoscopy due to inadequate bowel preparation at baseline are lacking, but the rate observed in our cohort appears consistent with that reported in the literature for post-colonoscopy consultations, which ranges from 8.4% to 14%. 17 18 19

The risk of missing lesions is higher in cases of inadequate bowel preparation. 4 Our study revealed an ADR of 31.5% on repeat colonoscopy. Indeed, 2 CRC (1.5%) were missed on index colonoscopy and detected on repeat procedure. Although this study is not powered to draw definitive conclusions on pathology findings, these results align with previous data. A large meta-analysis of tandem colonoscopies showed a 26% missed adenomas. 20 Another study reported an ADR of 45.3% and CRC detection of 1% in repeat colonoscopies following inadequate preparation. 21

Ensuring the return of individuals with inadequate bowel preparation for repeat colonoscopy is a challenging task, influenced by several contributing factors. In our study, although the implementation of a written recommendation led to significant improvement, 45.6% of patients remained lost to follow-up within 1 year. A recent study reported that nearly half of the patients undergoing endoscopic resection of colorectal polyps ≥20 mm were also lost to follow-up, 22 suggesting that even in high-risk populations, additional barriers compromise appropriate surveillance. This highlights the need for further action and reflects the multifactorial complexity of optimizing follow-up after inadequate bowel preparation. Interventions such as nurse-led educational telephone calls prior to colonoscopy have demonstrated efficacy in reducing patient no-show rates. 23 24 Currently, international guidelines do not define a minimum standard for adherence to follow-up recommendations due to insufficient evidence. 6 Our study may help establish quality thresholds and guide future improvements.

Multivariable analysis revealed that providing a written recommendation was the strongest factor associated with completion of repeat colonoscopy within 1 year (OR 3.40). This finding addresses an important gap in literature and supports a simple, scalable strategy to enhance the effectiveness of colonoscopy. Incomplete index colonoscopy (OR 2.56) and a surveillance indication (OR 2.77) were also significantly associated with higher compliance. An incomplete procedure is intuitively understood as inconclusive by both physicians and patients, and surveillance patients are more aware of the need for follow-up due to prior pathology. 25

Our study has several strengths. (1) This is the first study to directly assess the impact of a written recommendation on repeat colonoscopy completion within 1 year in case of inadequate bowel preparation, outside screening programs. (2) Unlike previous studies, we prospectively recruited participants for the intervention group allowing us to collect factors associated with completion of repeat colonoscopy. (3) We used the Boston Bowel Preparation Scale, the most thoroughly validated and recommended scale for assessing bowel cleanliness. 26 (4) We included patients undergoing colonoscopy for a variety of indications beyond screening, allowing a better generalization of our findings in real clinical practice.

We also acknowledge several limitations. This was a single-center, nonrandomized study with a historical control group, which limits the generalizability of the findings and precludes definitive causal inference. Although no major changes in healthcare organization, colonoscopy scheduling practices, or the overall bowel preparation strategy were identified during the study period, the use of cohorts from different time periods means that residual temporal confounding cannot be fully excluded. Therefore, the results should be interpreted with caution and confirmed in prospective multicenter studies.

In conclusion, providing a written recommendation after inadequate bowel preparation improves completion of repeat colonoscopy within 1 year. Implementing a mandatory field in the colonoscopy report is an effective measure to ensure guideline-concordant follow-up recommendations.

## Data Availability

The underlying research data of this article are not publicly available due to patient privacy and data protection regulations. The data are available from the corresponding author on reasonable request.

## Declaration of GenAI Use

The authors declare the use of generative AI exclusively to support manuscript preparation by assisting with translation, reformatting, and improving the clarity, grammar, fluency, and readability of the English text. The tool used was ChatGPT (GPT-5.5, OpenAI, 2026). In accordance with the GAIDeT taxonomy (2025), these tasks were delegated to the generative AI tool under full human supervision.

All AI-assisted outputs were critically reviewed, verified, and revised by the authors to ensure their accuracy and appropriateness. The authors retain full responsibility for the final manuscript. The generative AI tool is not listed as an author and bears no responsibility for the final work.

## Supplementary Material

Ergänzendes Material Supplementary Material
