# Supplementary material for: Written Recommendation to Repeat Colonoscopy within 1 Year after Inadequate Bowel Preparation: A Simple and Highly Effective Intervention
Source: Endosc Int Open. 2026 Oct 16;14:a29737245. doi: 10.1055/a-2973-7245 (PMC13679638; doi:10.1055/a-2973-7245)
Supplement: Supplementary file 1 [file 10-1055-a-2973-7245_29748441.pdf]

Supplementary material: Configuration of a standardized recommendation tab in the Endobase system.

Authors´ names: Marta Antón Arnal, Nadine Grima Sopesens, Agustín Seoane Urgorri, Xavier Duran Jorda, Marc Batlle Garcia, Miriam Parrilla Carrasco, Eva Pont Feijoo, Anna Mañé Bosch, Jordi Muñoz Galito, Faust Riu Pons, Josep Maria Dedeu Cuscó, Xavier Bessa Caserras, Luis Barranco Priego, Miguel Ángel Pantaleón Sánchez.

Title of paper: Written recommendation to repeat colonoscopy within 1 year after inadequate bowel preparation: a simple and highly effective intervention.

To standardise clinical recommendations following colonoscopies with inadequate bowel preparation, a dedicated text block was created within the Endobase reporting platform. In the “Observation” tab a new entry was created under the category “Recommendation for inadequate preparation” (Supplemental Figure 1). The recommendation text was formulated as follows: *“Due to inadequate bowel preparation, the examination is considered invalid. A repeat colonoscopy is recommended within one year.”* The text block was inserted into patient reports with a single action (Supplemental Figure 2).

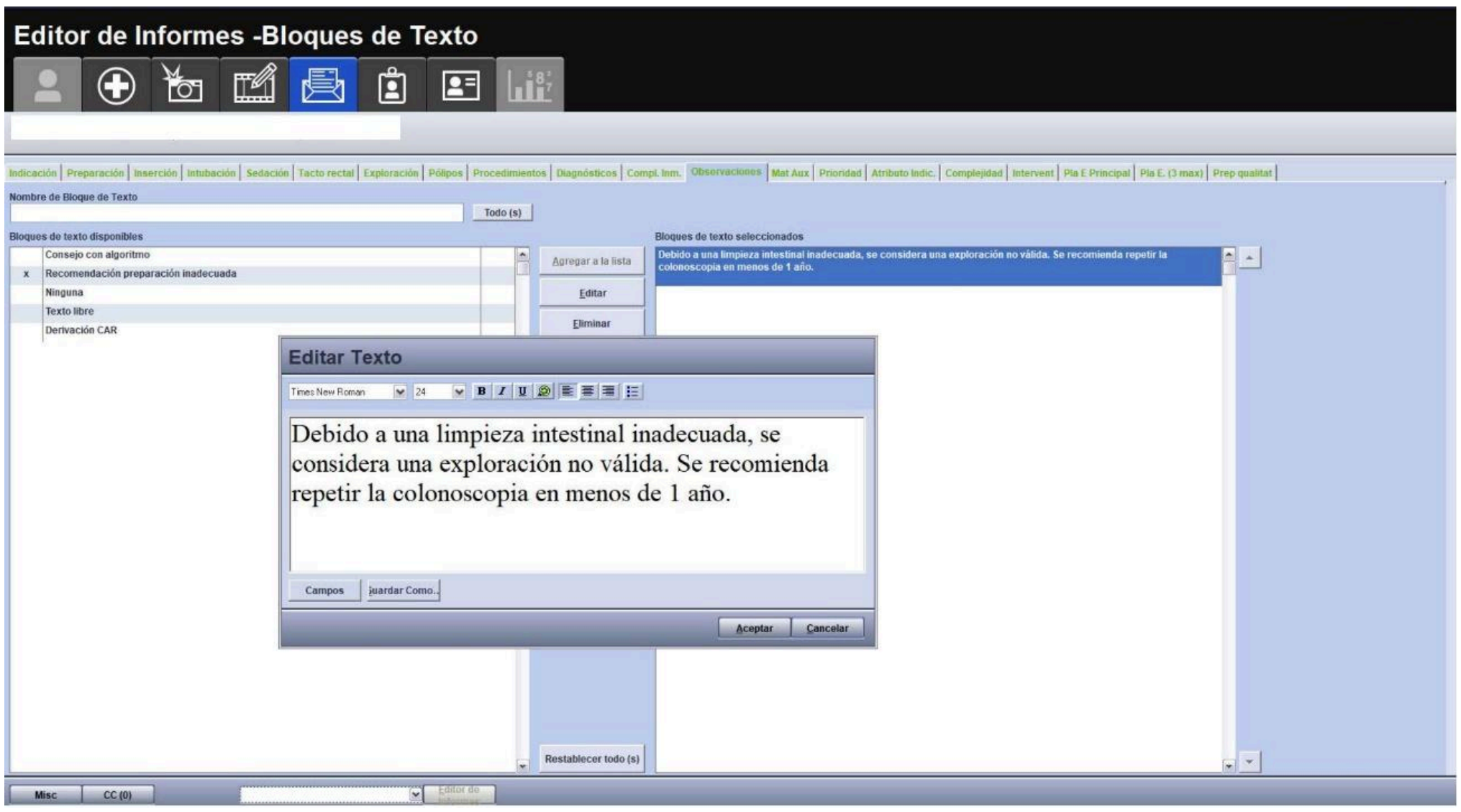

Supplemental Figure 1: Mandatory tab and text block in case of inadequate bowel preparation

Thieme

COLONOSCOPIA

Indicación: Dolor abdominal.

Preparación: Clasificación Boston de colon derecho: 0
Clasificación Boston de colon transverso: 1
Clasificación Boston de colon izquierdo: 1
NA: No aplicable
0: Prácticamente no se ve nada por los restos fecales
1: Sólo se puede ver una parte de la superficie mucosa por restos fecales olíquido opaco
2: Se puede ver la mayor parte de la superficie mucosa, por pequeñas contidades de restos
3: Se ve completamente la superfície mucosa.

Sedación: 200mg de propofol

Inserción:Se explora hasta lo que impresiona ser el ángulo hepático por mala preparación.

Exploracion:
Abundantes restos fecales parcialmente aspirables en todo el marco cólico que impiden una adecuada visualización de la mucosa. Dado el riesgo de yatrogenia, se suspende el procedimiento.

Procedimientos: -

Diagnósticos: Preparación inadecuada. Colonoscopia incompleta.

Compl. Inm.: No se observan. **Complejidad**: COM2
Intervencionismo: INT 2

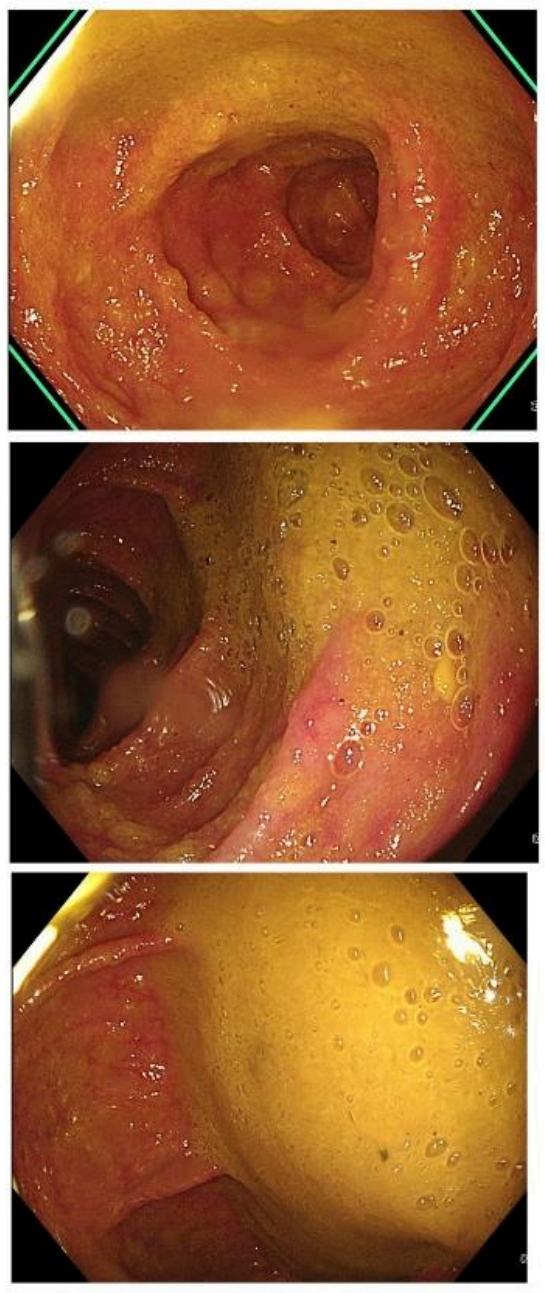

Observaciones: Debido a una limpieza intestinal inadecuada, se considera una exploración no válida. Se recomienda repetir la colonoscopia en menos de 1 año.

Supplemental Figure 2: Report example including written recommendation.
